# Temperature-Controlled Syngas Production via Electrochemical CO_2_ Reduction on a CoTPP/MWCNT Composite in a Flow Cell

**DOI:** 10.1021/acsaem.2c02873

**Published:** 2022-12-22

**Authors:** M. Noor Hossain, Reza Khakpour, Michael Busch, Milla Suominen, Kari Laasonen, Tanja Kallio

**Affiliations:** Department of Chemistry and Materials Science, Aalto University School of Chemical Engineering, P.O. Box 16100, EspooFI-00076 AALTO, Finland

**Keywords:** CO_2_ reduction, flow cell, syngas, molecular catalysts, temperature influence

## Abstract

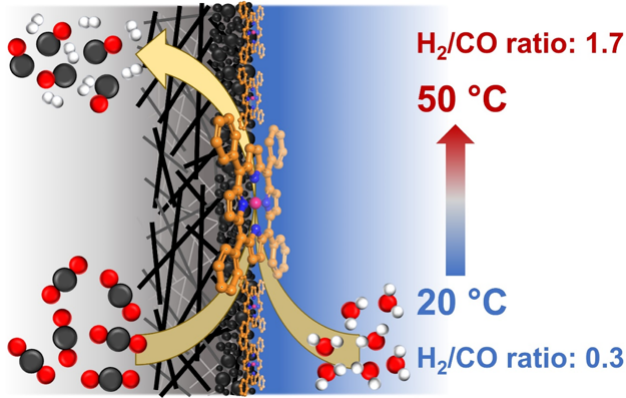

The mixture of CO and H_2_, known as syngas, is a building block for many substantial chemicals and fuels. Electrochemical reduction of CO_2_ and H_2_O to syngas would be a promising alternative approach for its synthesis due to negative carbon emission footprint when using renewable energy to power the reaction. Herein, we present temperature-controlled syngas production by electrochemical CO_2_ and H_2_O reduction on a cobalt tetraphenylporphyrin/multiwalled carbon nanotube (CoTPP/MWCNT) composite in a flow cell in the temperature range of 20–50 °C. The experimental results show that for all the applied potentials the ratio of H_2_/CO increases with increasing temperature. Interestingly, at −0.6 *V*_RHE_ and 40 °C, the H_2_/CO ratio reaches a value of 1.2 which is essential for the synthesis of oxo-alcohols. In addition, at −1.0 *V*_RHE_ and 20 °C, the composite shows very high selectivity toward CO formation, reaching a Faradaic efficiency of ca. 98%. This high selectivity of CO formation is investigated by density functional theory modeling which underlines that the potential-induced oxidation states of the CoTPP catalyst play a vital role in the high selectivity of CO production. Furthermore, the stability of the formed intermediate species is evaluated in terms of the *p*K_a_ value for further reactions. These experimental and theoretical findings would provide an alternative way for syngas production and help us to understand the mechanism of molecular catalysts in dynamic conditions.

## 1 Introduction

**Scheme 1:**
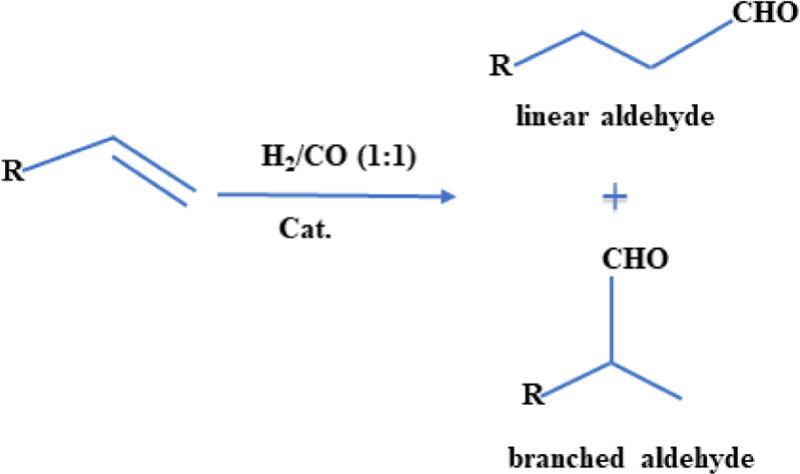
General Reaction Principle of Allyl Group and Syngas in the Presence of Catalysts to Produce Linear or Branched Aldehydes

Anthropogenic activities generate more than 30 billion tons of CO_2_ annually, and part of this gas accumulates in the atmosphere and contributes to the increase of atmospheric CO_2_ concentration, which has reached a record high level of ca 417 ppm recently.^1,2^ Among all the emission sources, chemical industries play a major role.^3^ For a sustainable future, actions are required to lower the atmospheric CO_2_ concentration and cut the emission. One promising approach is to use industrial CO_2_ waste streams as a feedstock for chemicals and fuel production as the conventional fossil fuel-based feedstocks need to be replaced with more environmentally friendly techniques. In this context, electrochemical reduction of CO_2_ and H_2_O (eCO_2_R) to syngas (mixture of H_2_ and CO) is a promising and potentially viable approach to convert CO_2_ waste streams into valuable commodity chemicals and fuels utilizing the Power-to-X (P-to-X) concept.^4^ Syngas is known as a building block for various high-value chemicals produced industrially, including alcohols, methane, dimethyl ether (DME), oxo-alcohols, ethylene, ethylene glycol, and many more.^5^ Currently, production of syngas utilizes environmentally detrimental techniques, for instance, coal gasification^6^ and steam reforming of fossil fuels or natural gas.^7^ These techniques are also among the major sources of industrial greenhouse gas emissions.^5^ Therefore, the eCO_2_R to syngas approach can provide the society with multiple benefits, such as, cutting the CO_2_ emissions by utilizing unavoidably emitted CO_2_ waste gas streams, for example, from steel production plants^8^ and the cement industry.^9^ Controlling the purity and H_2_/CO ratio of syngas is important for various downstream processes in the chemical industry, although the required ratios differ.^10^ For instance, H_2_/CO ratio of 1.0–1.2 is required for hydroformylation,^11^ such as the reaction depicted in Scheme 1.

The H_2_/CO ratio can be controlled by catalyst design. Numerous catalyst materials including metal alloys, metal nanoparticles, and molecular catalysts have been developed and investigated for the production of syngas via eCO_2_R in aqueous media.^12−17^ To obtain a desirable ratio in syngas, a bifunctional electrocatalyst consisting of Au and Co has been investigated by Michael et al.^18^ In their study, a H_2_/CO ratio of 0.6–2.1 was obtained in an aqueous environment. Varying amounts of Au nanoparticles loaded on a titanate nanosheet (TiNS) substrate have been investigated by Filipe et al. to obtain the desired syngas ratio.^19^ They have found that with the decrease of the amount of Au nanoparticle loading on the TiNS substrate, the H_2_/CO ratio increases. Along with the catalyst design, most of the approaches have also focused on investigating the applied potential for controlling the syngas H_2_/CO ratio.^20−22^

Molecular catalysts supported on highly conductive carbon materials have been investigated mainly for CO production. For instance, Fe, Co, and Ni molecular catalysts supported on carbon show >90% selectivity for CO production in aqueous electrolyte media in a so-called H-cell.^23−25^ The potential-dependent eCO_2_R to CO conversion has been investigated in a flow-cell configuration on a ligand-modified cobalt phthalocyanine/carbon nanotube (CoPC/CNT) composite and on Fe-porphyrin and resulted in FE >94% for CO formation.^26,27^ Interestingly, syngas production on molecular catalysts has drawn less attention, though few works have been performed either in an H-cell^28^ or a three-electrode cell^29^ on Fe-porphyrin-based metal–organic framework (Fe-MOF) catalysts in aqueous media. Cobalt tetraphenyl porphyrin (CoTPP) and its derivatives supported on carbon black also show >80% selectivity for CO production in the potential range from −0.5 V_RHE_ to −0.65 V_RHE_ in flow conditions.^30^ However, the current density shows a decreasing trend at −0.65 V_RHE_ during constant potential electrolysis. The catalyst support is crucial in terms of, for example, electronic conductivity, chemical and mechanical stability, and surface area;^31^ in this sense, temperature-controlled eCO_2_R to syngas production in a flow cell on CoTPP supported on carbon nanotubes (MWCNTs) has remained unexplored.

Indeed, temperature could affect the eCO_2_R by various ways such as increasing the pH and the conductivity of the electrolyte and decreasing the concentration of dissolved CO_2_, surface tension, viscosity of the electrolyte, and the adsorption strength of the intermediate species.^32−35^ Increasing temperature has been shown to switch the competition between the eCO_2_R and the hydrogen evolution reaction (HER). Product selectivity during the eCO_2_R on Cu has been shown to be largely affected by temperature, for example, CH_4_ and C_2_H_4_ production is favored at lower temperatures, while H_2_ is dominant at higher temperatures in an H-cell.^33^ Also, CO formation selectivity has been observed to decrease, while that of H_2_ increased with temperature increment in a flow-cell configuration on tin oxide and Ag catalysts, respectively.^34,36^ A highly selective eCO_2_R to the CO catalyst can strongly suppress the HER which inevitably takes place on the cathode according to reaction 1 when an aqueous electrolyte is used as the reaction medium. Applying temperature can reduce the degree of suppression of HER which would allow more H_2_ production along with eCO_2_R to CO according to reaction 2.^32^ Hence, besides the catalyst design tactics, applying temperature could be a fundamentally interesting approach to produce syngas with controlled H_2_/CO ratios.
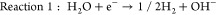

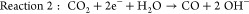


Herein, we have investigated temperature-controlled syngas production by eCO_2_R in a flow cell on a composite comprising cobalt tetraphenylporphyrin supported on multiwalled carbon nanotubes (CoTPP/MWCNTs). To the best of our knowledge, systematic investigation of low-temperature-controlled syngas production has not been performed in a flow cell on CoTPP catalysts. Our results indicate that the composite is highly selective for CO at 20 °C. Interestingly, this selectivity is altered with increasing temperature in the range of 20–50 °C, resulting in an increase of the syngas H_2_/CO ratio. To gain insight on the mechanism, the origin of high selectivity of the composite toward CO formation is investigated through DFT modeling. These results underline that at −0.8 and −1.0 *V*_RHE_, the eCO_2_R to CO becomes barrierless compared to its counterpart HER. In addition, the potential-induced oxidation state of the Co atom of the CoTPP molecule triggers the catalytic activities for CO formation. Moreover, the introduction of the *p*K_a_ value in addition to the activation barrier reveals the stability of the formed intermediate species such as Co–H for HER and Co–COOH for eCO_2_R. This approach combining experimental and theoretical studies is beneficial for both investigating the production of syngas and for understanding the eCO_2_R mechanism.

## 2 Experimental Section

### 2.1 Electrode Preparation

A CoTPP-to-MWCNT ratio of 6:1 was chosen for these experiments based on earlier optimization.^37^ Detailed description of the synthesis and structural characterizations of the CoTPP/MWCNT (6:1) composite can be found elsewhere.^37^ Briefly, 50 mg of MWCNTs (9 nm diameter, >95% carbon, Nanocyl) was dispersed in 100 mL of 2 mM HCl (Merck) aqueous solution. 300 mg of CoTPP (dye content 85%, Sigma-Aldrich) was dispersed separately in 92 mL of 2 mM HCl (Merck) solution with the help of 8 mL of DMF (99.8%, Sigma-Aldrich). Both dispersions were sonicated for 1 h. After mixing, the MWCNT and CoTPP dispersion was sonicated again for 1 h followed by stirring for 48 h. Then, the supernatant solution was thrown away, and the precipitate was centrifuged for 1 h at 4000 rpm at 20 °C. This step was repeated multiple times. Finally, the precipitate was washed with Milli-Q water and freeze-dried for 24 h.

In all prepared electrodes for the flow-cell experiments, the CoTPP/MWCNT composite loading was kept at 1 ± 0.1 mg/cm^2^. An appropriate amount of the composite was weighed and dispersed in a mixture with 300 μL of pure ethanol and 150 μL of Milli-Q H_2_O. Subsequently, 5 wt % Nafion (Sigma-Aldrich) solution was added in the ink to obtain a 30 wt % Nafion ionomer content. The ink was sonicated for ca. 10–15 min and then stirred for 20–30 min and subsequently sprayed on 10.2 cm^2^ microporous carbon paper (Sigracet 25 BC) gas diffusion electrode (GDE) by employing an air brush. Before deposition of the composite, the GDE was cleaned with ethanol and dried in a vacuum oven for 2 h at 60–70 °C and weighed. After spray painting, the electrode was dried again in a vacuum oven at 60–70 °C for 2–3 h to remove the solvents. The weight difference before and after spraying gives the amount of the loaded composite on the GDE.

### 2.2 Chronoamperometry and Product Analyses

**Figure 1:**
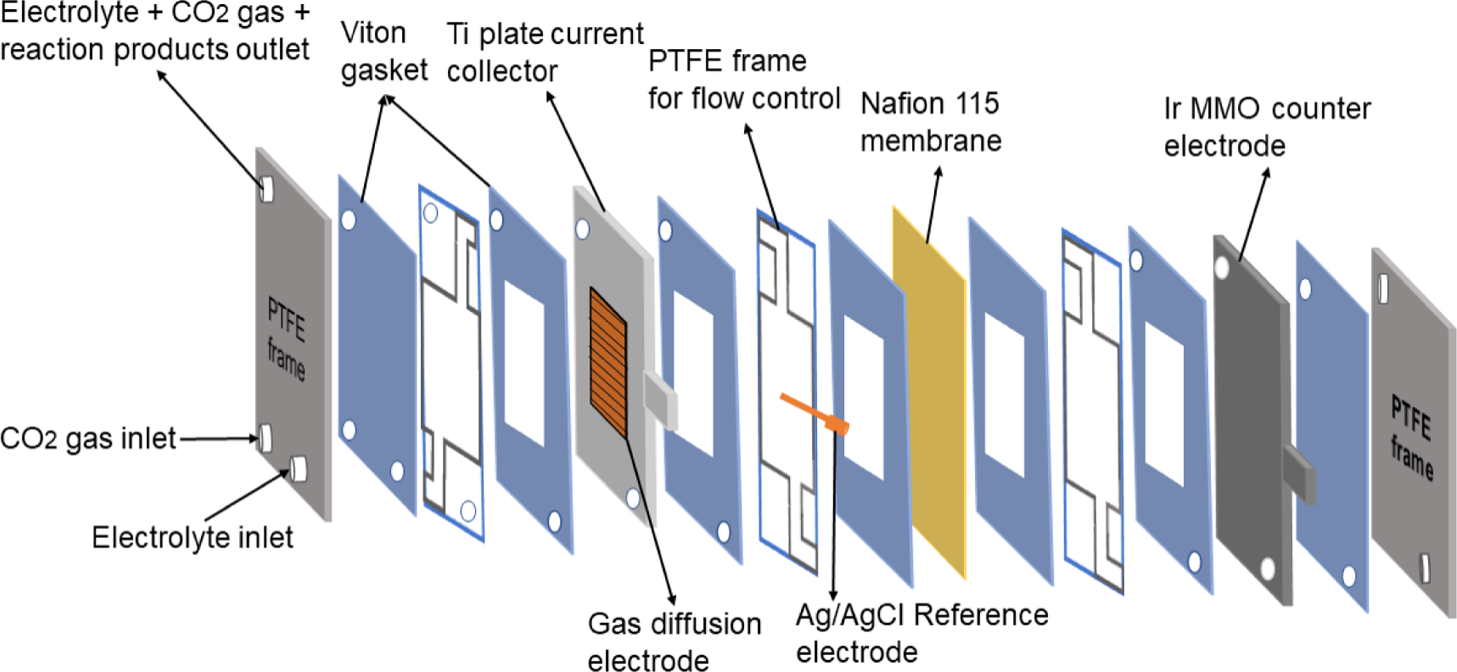
Schematic view of the flow cell.

A custom-made electrochemical flow cell was employed to perform constant potential electrolysis. The schematic view of the flow cell consisting of a cathodic and anodic compartment separated with a pretreated Nafion 115 membrane is shown in Figure 1. The working electrode is mounted on a Ti current collector plate. An Ir mixed metal oxide frame (Ir MMO, Electrocell A/S) and a 1 mm leak-free Ag/AgCl electrode (Innovative instruments) were used as the counter and reference electrodes, respectively. Polytetrafluoroethylene (PTFE) flow frames were used to direct the electrolyte and gas flow during the operation. Viton gaskets were used between the flow frames and current collectors.

0.1 M KHCO_3_ served as an electrolyte. A peristaltic pump was used to circulate the electrolyte at 15 mL/min in the front side of the working and counter electrodes. N_2_ and CO_2_ were purged behind the GDE in a separate gas compartment and collected into the catholyte outlet stream. Chronoamperometry (CA) was performed in a CO_2_ environment by employing an Ivium potentiostat (Iviumstat.XRi, Ivium technologies). The applied potentials were converted to the potential of the reversible hydrogen electrode (RHE) by employing eq 1.
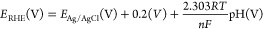
1where *E*_Ag/AgCl_(V) is the applied potential, *R* is the universal gas constant (8.314 J K^–1^ mol^–1^), *T* is the applied temperature (in Kelvin), *n* is the number of electrons transferred in the reaction, and *F* is the Faraday constant (96485 C mol^–1^).

Manual *iR* correction was performed. To compensate for the *iR* drop, electrochemical impedance spectroscopy (EIS) was employed to determine the ohmic resistance. The optimum frequency was chosen by applying EIS frequency ranging from 1 kHz to 1 Hz. The measured Ohmic resistance was then multiplied by the current obtained in cathodic cyclic voltammetry (CV) measurement, and the potential was corrected according to eq 2.
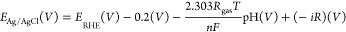
2

All potentials reported here are *iR* corrected. Before CA measurements, N_2_ was purged for ca. 30 min to remove O_2_ from the reaction environment, and then CV measurement was carried out. After that, CO_2_ was purged for 30–40 min to saturate the electrolyte with CO_2_, and CV measurement was repeated. CA measurements were performed at −0.6 *V*_RHE_, −0.8 *V*_RHE_, and −1.0 *V*_RHE_ at selected temperatures (20, 30, 40, and 50 °C). The pH of the CO_2_-saturated electrolyte was measured at each temperature equaling 6.76, 6.86, 7.17, and 7.23 for 20, 30, 40, and 50 °C, respectively. The temperature of the cell was controlled by placing catholyte and anolyte reservoirs in a temperature-controlled water bath. The cell temperature was determined from catholyte and anolyte outlet streams by a thermometer. During CA, CO_2_ was continuously purged into the cathode through the GDE with a flow rate of 19 SCCM to keep the constant supply of the reactant gas in the electrode/electrolyte interface. The catholyte reservoir was connected to a micro gas chromatograph (mGC, Agilent 990). Between the catholyte reservoir and the mGC, an ice trap was used to lower the humidity of the outlet gas stream to protect the mGC columns. The effluent gases were passed through a flow meter before entering the mGC. The gaseous products were analyzed with the mGC equipped with CP-MoleSieve 5A and CP-PoraPLOT U 10 m long columns and micro-machined thermal conductivity detectors (TCD). The permanent gases including CO, H_2_, and N_2_ were separated in the Mol Sieve 5A column, while the hydrocarbons, for example, CH_4_, C_2_H_4_, and CO_2_ were separated in the PoraPLOT U column. Liquid products were analyzed using a high-performance liquid chromatograph equipped with an Aminex 87H column and a refractive index (RI) detector.

The partial current density of the gaseous products was calculated by using eq 3.
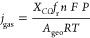
3where *j*_gas_ is the partial current density of gases, *X*_CO_ is the mole fraction of the gases, *f*_r_ is the flow rate of the effluent gas in L/s, *n* is the number of electrons, *F* is the Faraday constant, *P* is the atmospheric pressure, *A*_geo_ is the geometric surface area of the electrode, *R* is the universal gas constant, and *T* is the temperature (in Kelvin). The mole fraction, *X*_CO_, is calculated by using eq 4.
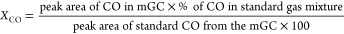
4

Faradaic efficiency (FE) was calculated by using eq 5.
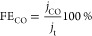
5where FE_CO_ is the FE of CO and *j*_t_ is the total current density.

The FE of the liquid products was calculated by using eq 6.
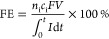
6where *n*_i_ is the number of electrons required to produce the *i*_th_ product, *c*_i_ is the concentration of the product obtained from HPLC analysis, *F* is the Faraday constant, *V* is the volume of the catholyte, and *I* is the average current during CA measurements.

The turn-over frequency (TOF) calculation was performed by using the following formula
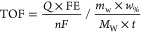
7where *Q* is the total charge during chronoamperometry, FE is the Faradaic efficiency, *F* is the Faraday constant, *n* is the number of electrons exchanged for the product formation, *m*_w_ is the mass of the composite loaded on the GDE, *w*_%_ is the weight % of the catalyst (e.g., Co for CoTPP) from XPS, *M*_W_ is the molecular weight of the catalyst, and *t* is the time used to express the TOF unit.

The rate of electrochemical CO and H_2_ production is calculated by following the Arrhenius equation
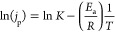
8where *j*_p_ is the partial current density of the reaction products, *K* is a constant, *E*_a_ is the activation energy, *R* is the universal gas constant, and *T* is the temperature.

### 2.3 Computational Details

A triple ξDef2-TZVP^38^ basis set and the M06^39^ functional as implemented into Gaussian 16 Rev C.01 [4] in combination with an ”ultrafine” grid size were used to optimize the structures of all compounds. This functional has been reported to perform the best for transition-metal-doped porphyrins considered in this study.^40^ Solvation effects were included through the implicit SMD solvation model.^41^ Redox potentials of proton-coupled electron-transfer (PCET)^42^ steps were computed using the computational normal hydrogen electrode which uses H_2_ in the gas phase as discussed in ref (43). Electron-transfer potentials were calculated using the effective absolute potential method which relies on the absolute potential of the SHE computed for the used computational setup.^44^*p*K_a_ values were obtained using the isodesmic method in combination with formic acid as a reference compound and an appropriate scaling relationship to correct for the shortcomings of the implicit solvation model. Following an earlier work, eq 8 was used as correction.^45^
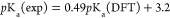
9

Calculations of transition-state (TS) structures were performed using the quadratic synchronous transit (QST3) method. Owing to significant instabilities in the forces, this method failed for several structures. In these cases, manual potential energy surface (PES) scans along the relevant reaction coordinates were performed. Also, barrierless transitions were confirmed by PES scans. All reported TS structures were tested for the presence of exactly one imaginary frequency along the reaction coordinate. Ground states were assumed to be converged if no imaginary frequencies were present.

The CoTPP/MWCNT composite was estimated by a Co-porphyrin (CoTPP) molecular model. The most favorable spin states were determined through explicitly testing of all reasonable spin states. This small model system was chosen as it allows for the use of high-level DFT methods while retaining the central structural features of the active site of the catalyst. Moreover, using a molecular system we can easily model different oxidation states by charging the system. If we would use periodic boundary conditions, the charges could interact if the system is too small, resulting in wrong total energies. Potentials have been calculated based on an RHE reference at pH = 7. (For more information regarding computations, please refer to the Supporting Information.)

## 3 Results and Discussion

### 3.1 Electrocatalytic Activities of the Composite

**Figure 2:**
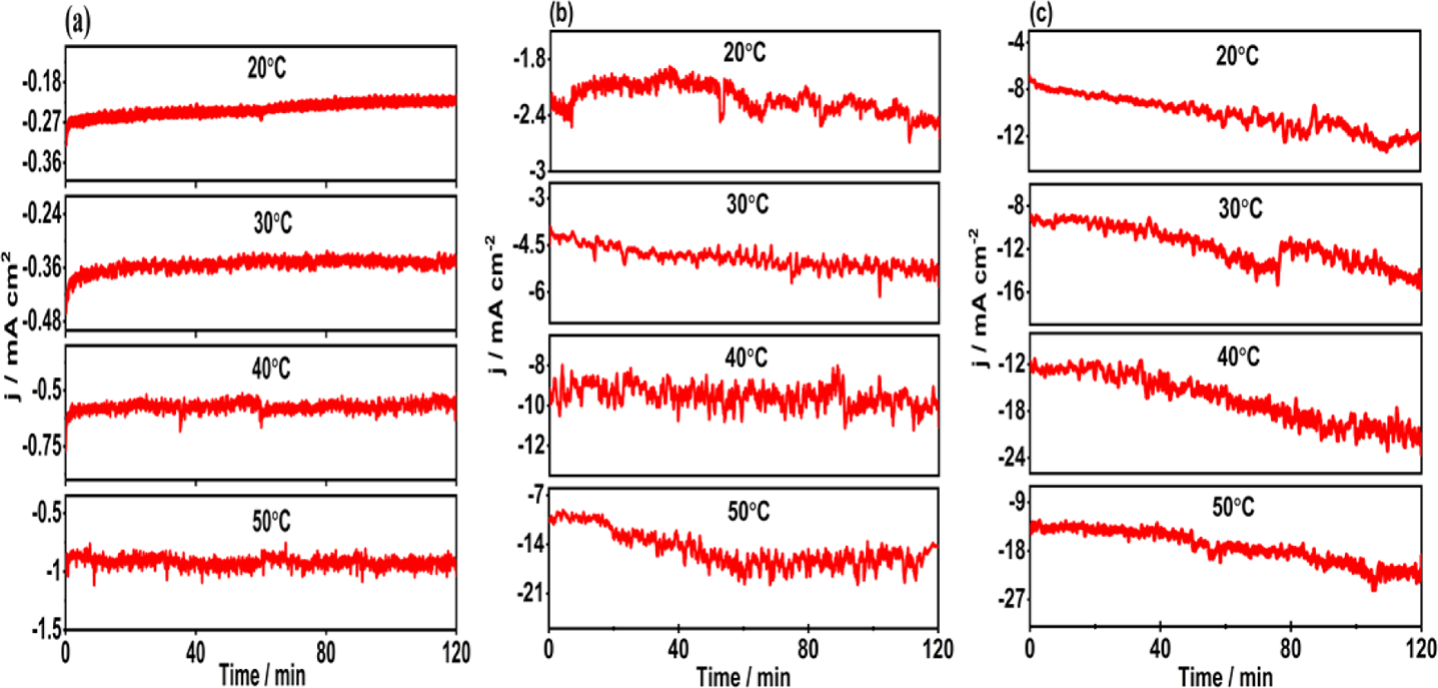
Chronoamperometry curves for the CoTPP/MWCNT composite at four studied temperatures of (a) −0.6 *V*_RHE_, (b) −0.8 *V*_RHE_, and (c) −1.0 *V*_RHE_.

To investigate product distribution on the CoTPP/MWCNT composite, electrolysis was performed in a flow cell at a constant potential, while CO_2_ was continuously supplied in the backside of the GDE (Figure 1). The gas flow generates a convective force, helping CO_2_ molecules to reach the electrode/electrolyte interface at the three-phase boundary where the electroreduction reaction occurs. In the investigated single-atom type electrocatalyst, Co^2+^ atoms in the CoTPP molecule are isolated by tetraphenylporphine (TTP) ligands and are expected to function as the active sites. At sufficiently negative potentials, CO_2_ molecules should adsorb on the Co centers and convert to CO, which is then released from the surface before further reduction,^46^ as will be shown later with DFT modeling. Catalytic activities of the composite have been evaluated by CA measurement for 2 h, and the results are presented in Figure 2**.** The curves show two distinguishable features of the total current density (*j*_T_), the increment over the time and the oscillation. Figure 2a shows that during the 2 h long measurements at −0.6 *V*_RHE_, the activity of the composite remains rather steady except at 50 °C where the activity slightly increases, indicated by the increase in *j*_T_ over time.

Applying more negative potentials enhances the overall catalytic activities of the composite as indicated by increasing *j*_T_ for all studied temperatures presented in Figure 2b,c. At −0.8 *V*_RHE_ (Figure 2b) and at −1.0 *V*_RHE_ (Figure 2c), the *j*_T_ increased over time in all four temperatures studied. This may occur either from demetallation of the molecular catalysts or increasing wettability of the electrode. Although it is hard to completely exclude demetallation of the catalysts as a possible reason for current density increment over time, there is an alternative explanation based on the scanning electron microscopy (SEM) images, X-ray diffraction (XRD) pattern, CV measurement, time vs mol % of product formation analysis, 1 h CA measurement on pristine MWCNTs at −0.8 *V*_RHE_ in a CO_2_ environment, and earlier studies on catalyst demetallation. The SEM images of fresh and used electrodes (see Figure S2) show similar morphologies. The XRD analysis performed for a fresh and a used composite electrode, pristine CoTPP, and MWCNTs (Figure S3) can reveal structural changes in the used electrodes as shifting of the major 2θ peaks or change in the peak intensity ratios.^47^ In the XRD pattern of the composite, the 2θ peaks at 20 and 26.5° correspond to the main peak of CoTPP and MWCNT, respectively. The intensity ratio of these two peaks is 3.9 for both fresh and used electrodes. In addition to this, other 2θ values of the fresh and used composite electrodes show similar characteristics indicating that major changes do not take place on the catalyst under the selected reaction conditions. In case of catalyst demetallation, the most pronounced indication would be the decrease of current density over time due to increase of resistance.^48^ To investigate this, CV measurements were performed before and after the 2 h CA measurement at −0.8 V_RHE_ (see Figure S4). In accordance with the CA measurements, the CV measurements show increasing current density after 2 h of CA. Demetallation may also cause a decrease in the formation of one of the products.^49^ As discussed below in more detail, only CO and H_2_ are detected as the major products, and their time versus mol % curves at −0.8 *V*_RHE_ and −1.0 *V*_RHE_ (see Figures S5 and S6) show that the formation of both products increase over time.

**Figure 3:**
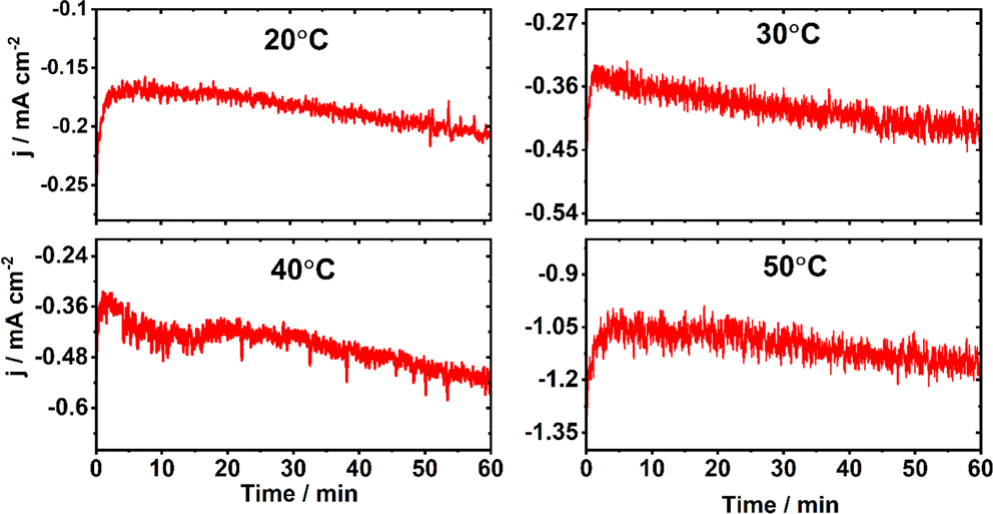
CA measurement at −0.8 *V*_RHE_ on pristine MWCNTs in a CO_2_ environment.

To investigate the contribution of MWCNTs on the overall electrode activity, 1 h CA measurement on pristine MWCNTs was carried out in the flow cell at −0.8 *V*_RHE_ at the four studied temperatures (Figure 3). These measurements show a similar trend of current density increment over time. Earlier studies have proved that in the case of demetallation, catalysts promote the HER rather than eCO_2_R.^48,49^ For example, Hailiang et al. showed that due to demetallation of CoPC/MWCNT hybrid catalysts, the selectivity of CH_3_OH formation decreases from the FE of 44 to 0.6%, while the FE of H_2_ increases to 80%.^48^ In this case, current density also shows a decreasing trend. Thus, all experimental evidence and earlier findings support the above discussion and suggest that catalyst demetallation is unlikely.

The other possible reason for the current density increment is the increased wettability of the electrode resulting from gradual reduction of hydrophobicity of the electrode. The reduced hydrophobicity intrinsically causes perspiration of the electrolyte,^50,51^ which may contribute to increase the rate of hydrogen formation. Notably, electrode wettability strongly depends on the applied potential.^52,53^ This is also evident from the CA measurement at −1.0 *V*_RHE_ where current density increment at −1.0 *V*_RHE_ is higher than at −0.8 *V*_RHE_. Likewise, a higher temperature also contributes to an increase in the wettability of the electrode during the operation which is evident from the CA measurement at 50 °C in Figure 2 and also from previous studies.^34,54^ Hence, during the CA measurements, the deactivation of the composite is unlikely which underlines the stability of the composite under reducing conditions. The oscillation of *j*_T_ is ascribed to the so-called bubble nucleation, growth, and detachment effect on the electrode surface. Whenever the produced gas bubbles occupy a fraction of the electrode surface, the bubbles block the electrode surface and induce a decrease of the current density. When the bubbles detach from the electrode surface, the current density starts to increase again.

**Figure 4:**
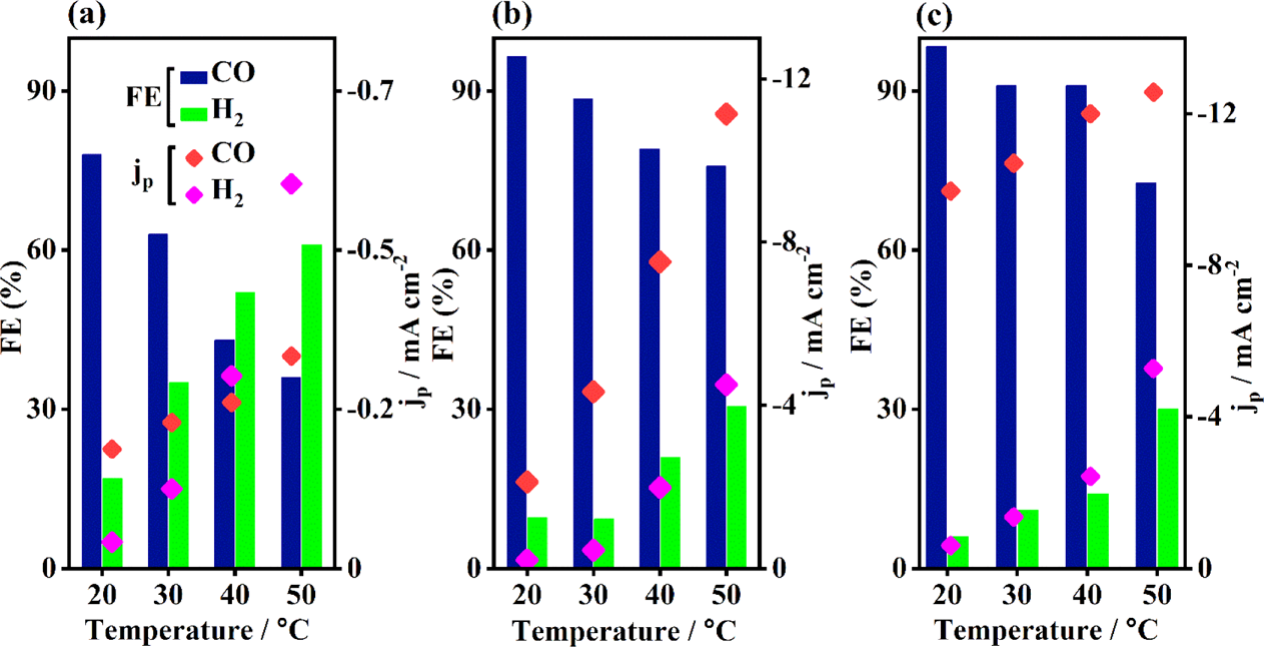
FEs and partial current densities for CO and H_2_ production on the CoTPP/MWCNT composite at four studied temperatures of (a) −0.6 *V*_RHE_, (b) −0.8 *V*_RHE_, and (c) −1.0 *V*_RHE_.

The FE of a reaction product is the input and output charge balance during the constant potential electrolysis, while partial current density (*j*_p_) represents the amount of products after a certain duration. The FEs and partial current densities of CO and H_2_ under different potentials and temperatures are presented in Figure 4. Figure 4a shows the FE and *j*_p_ of CO and H_2_ at −0.6 *V*_RHE_ at the studied temperatures. The highest FE of CO (ca. 78%) is obtained at 20 °C, while the FE of H_2_ is ca. 17%. At 30 °C, the FE of CO slightly decreases to 63%, while the FE of H_2_ increases to ca 35%. However, at 40 and 50 °C, the FE of H_2_ reaches 52 and 61%, respectively, while the FE of CO decreases to ca. 43 and 36%, respectively. These changes in the product distribution clearly show that H_2_ production is favored at the elevated temperatures. The influence of temperature is also observed in the partial current density for CO and H_2_. At 20 °C, the CO partial current density (*j*_CO_) is ca. −0.18 mA/cm^2^ and it increases to −0.32 mA/cm^2^ at 50 °C. Similarly, for H_2_ formation, the partial current density (*j*_H2_) increases to −0.58 mA/cm^2^ at the highest studied temperature, showing 10 times higher value compared to its current density at the lowest studied temperature. This indicates that temperature plays an important role during the eCO_2_R by also enhancing the reaction kinetics. It is noteworthy that neither other gaseous nor liquid products are detected at this potential.

The FE and *j*_p_ of the reaction products at −0.8 *V*_RHE_ are presented in Figure 4b. At this potential, the FE of CO reaches 96% at 20 °C, indicating that the composite is very selective toward CO_2_ to CO conversion under these conditions. However, the FE of CO decreases with increasing temperature also at this potential. Conversely, the FE of H_2_ is ca. 7% at 20 °C but 26% at 50 °C. Only HCOO^–^ is identified as the liquid product in low amounts as its FE is ca. 1%. Interestingly, *j*_p_ values of both CO and H_2_ increase with temperature. The *j*_CO_ at 20 °C is ca. −2 mA/cm^2^, while at 50 °C, it increases to ca. −11 mA/cm^2^. The *j*_H2_ at 20 °C is ca. −0.2 mA/cm^2^, whereas at 50 °C, it is −4.5 mA/cm^2^. Thus, it is clear that the rate of the reaction for both products at this potential increase with increasing temperature even more than at −0.6 *V*_RHE_. Applying a more negative potential of −1.0 *V*_RHE_ also results in a higher FE and *j*_p_. The FE and *j*_p_ of the reaction products at −1.0 *V*_RHE_ are presented in Figure 4c. The FE of CO reaches 98% at 20 °C, confirming that the composite is highly selective for CO_2_ to CO conversion also under these reaction conditions. Likewise, for the measurements at −0.8 *V*_RHE_, the selectivity toward CO formation decreases when the temperature increases from 20 °C to 50 °C. At the latter temperature, the FE of CO is ca.72%. The opposite trend is observed for H_2_ formation as the FE is ca. 6% at 20 °C and ca. 30% at 50 °C. For the only minor product, HCOO^–^, the FE is still less than 1%. Furthermore, the *j*_CO_ at the lowest studied temperature 20 °C is ca. −9 mA/cm^2^, while it increases to −12 mA/cm^2^ at the highest studied temperature. The *j*_H2_ at 20 °C is ca. −0.6 mA/cm^2^, while at 50 °C, it is −5.2 mA/cm^2^. The *j*_H2_ at −1.0 V_RHE_ is higher than the values at −0.6 *V*_RHE_ and −0.8 *V*_RHE_, indicating significant *j*_H2_ increases with increasing applied potential along with the temperature.

Interestingly, for all studied potentials, at 20 °C the FE of H_2_ and *j*_H2_ are quite low, but with increasing temperature both are increasing. This trend of H_2_ production suggests that at 20 °C, the HER presumably takes place on the MWCNT site which is supported by the 1 h CA measurement on the MWCNT electrode (see Figure 3) in a CO_2_ environment, producing H_2_ as only reaction product with a FE of ca. 100% at the four studied temperatures at −0.8 *V*_RHE_. This is consistent with the earlier studies where H_2_O may function as a H^+^ source.^55,56^ DFT analysis also shows that HER is unlikely to proceed on the Co active sites due to a high activation barrier (for details, see Section 3.4). However, at elevated temperature a fraction of Co active sites may participate in H_2_ production due to a decrease of the activation barrier. The opposite trend is observed for the CO generation which also suggests the Co participation for the HER at elevated temperatures.

In summary, the lower temperatures are favorable for eCO_2_R, while higher temperatures promote the H_2_ evolution reaction more. At more negative potentials, for example, −0.8 *V*_RHE_ and −1.0 *V*_RHE_, the composite is more selective for CO production at all studied temperatures, while at −0.6 V_RHE_ the selectivity for CO is quite low. The origin of these selectivity differences has been analyzed by DFT and discussed below in Section 3.4. Importantly, the temperature contributes to switching the selectivity between CO and H_2_ formation which can be used to produce varying ratios of H_2_/CO and is further discussed in the following section. The continuous increase of *j*_CO_ from lower to higher temperatures suggests that direct supply of CO_2_ behind the working electrode in these reaction conditions is sufficient. This is consistent with other works performed in flow-cell systems.^27,57^

### 3.2 Electrochemical Cell-Dependent Product Distribution

The product distribution of eCO_2_R strongly depends on the reaction conditions, which is seen by comparing our previous^37^ and current studies. In the previous study, an H-cell was used for constant potential electrolysis with the same electrolyte and composite loading on the electrode and the same temperature and potential range, that is, under similar conditions to the current study. In the H-cell, CH_3_OH and CO production is observed at all studied potentials and temperatures, whereas formate production is observed at −0.8 *V*_RHE_ and −1.0 *V*_RHE_ at all temperatures. Lower temperatures favor CH_3_OH formation in all studied potentials, while conversely higher temperatures favored H_2_ formation. CO formation is favorable at −0.6 *V*_RHE_ and −0.8 *V*_RHE_ in the temperature range 20–40 °C, while at −1.0 *V*_RHE_, it is only favorable at 20 °C. CH_4_ formation is observed at −1.0 *V*_RHE_ at 40 °C and 50 °C temperatures only.

As described above, the product distribution clearly shows potential and temperature dependency but is also affected by the measurement arrangements. In the current study, the only liquid product is formate which is detected at −0.8 V_RHE_ and −1.0 V_RHE_ with a low FE<1%. CO and H_2_ are detected at all temperatures and potentials as the major products, while CO is the main product except at the highest measured temperatures of 40 and 50 °C at −0.6 *V*_RHE_. The difference in the product distribution in comparison to the H-cell is suggested to rise due to the following reasons: (1) Mass transport. In the H-cell, a stagnant electrolyte layer is formed on the electrode surface because of adhesion between the electrode and solvent molecules. Sparely dissolved CO_2_ diffuses to the electrode surface, and CO_2_ together with the generated intermediate products can stay a longer time in the vicinity of the electrode because of the stagnant layer. Hence, the intermediates can react further on the electrode. In contrast, in the flow cell, the continuously forced CO_2_ gas flow removes the formed CO, regenerating the electrode surface, thus preventing potential further reactions. (2) In the H-cell electrode, the ionomer loading is 10 wt %, while in the flow cell, it is 30 wt %, and this hydrophobicity difference may cause the difference in CO_2_ adsorption and coverage on the catalyst surface.^58^ (3) The properties of the GDE, that is, porosity, surface area, PTFE loading, resistance, and so forth, are different in the H-cell and flow-cell electrodes prepared by using carbon cloth (GDL-CT, FuelCellsEtc) and carbon paper (Sigracet 25 BC, FuelCellsEtc), respectively. The GDE properties can combinedly affect the mass transport, conductivity, and active surface area of the composite materials.

### 3.3 Evaluation of Temperature Influence on Syngas and Intrinsic Activity of the Composite

**Figure 5:**
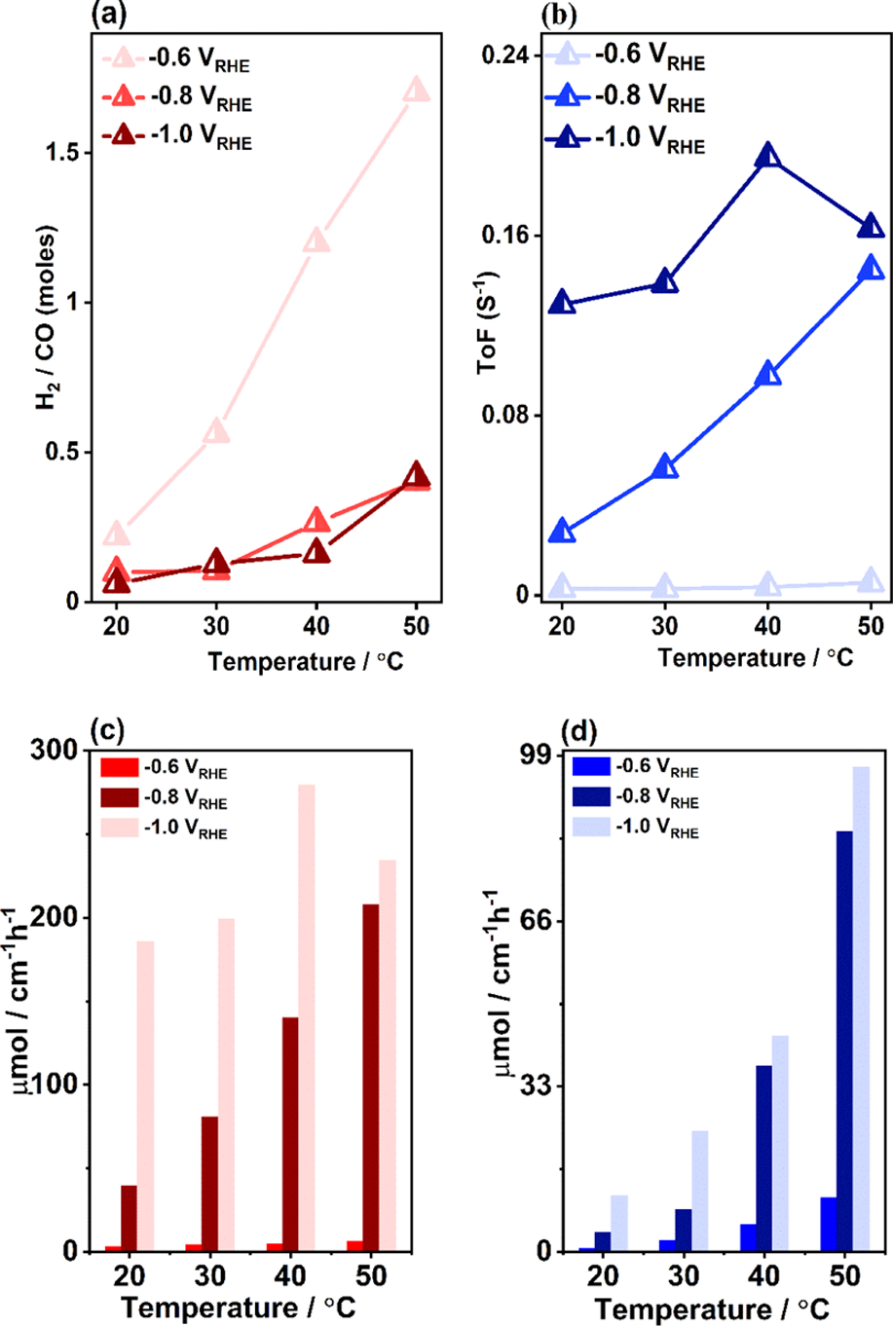
Temperature-controlled electrocatalytic activities of the composite. (a) Ratio of H_2_ and CO in the produced syngas, (b) TOF of CO, (c) CO production rate (μmol/cm^–2^ h^–1^), and (d) H_2_ production rate (μmol/cm^–2^ h^–1^) for all studied potentials and temperatures.

In case of syngas, the ratio between CO and H_2_ is clearly dependent on the studied temperature as shown in Figure 5a. Generally, syngas is the mixture of CO and H_2_ which is the building block for many industrially important chemicals. In the case of the Fischer–Tropsch synthesis of chemicals and fuels, the desired syngas H_2_/CO ratio is 0.3–4.^59^Figure 5a shows that at −0.6 *V*_RHE_, the H_2_/CO ratio is 0.3 at 20 °C but it increases to ca. 1.2 at 40 °C. The 1.2 H_2_/CO ratio is important for the synthesis of oxo-alcohols and aldehydes, for example, propionaldehyde, pentanols, 1-octanols, and many more.^11,60^ At 50 °C, it reaches ca. 1.70 which is close to the ratio 2 required for methanol synthesis from syngas. Hence, the increase of the ratio with temperature is a very important indication for achieving industrially important chemicals via eCO_2_R. The reaction temperature is undoubtedly controlling the formation of syngas also at the more negative potentials, although the syngas H_2_/CO ratio is much lower at these potentials than at −0.6 *V*_RHE_. At −0.8 *V*_RHE_, the syngas ratio increases from ca. 0.1 at 20 °C and reaches a maximum of 0.4 at 50 °C. At −1.0 *V*_RHE_, it increases from ca. 0.06 at 20 °C and reaches a maximum of 0.4 at 50 °C. Hence, for both −0.8 *V*_RHE_ and −1.0 *V*_RHE_ potentials, the H_2_/CO ratio at 50 °C could be utilized for the Fischer–Tropsch process.^18^

For the industrial application of syngas, the mixture needs to be completely free from CO_2_ gas. Such mixture could be achieved by cell engineering but is beyond the scope of the current study. Moreover, there is ongoing research on CO_2_ separation from syngas, for example, absorption and desorption by ionic liquids.^61^

The intrinsic catalytic activity of the composite is revealed by the TOF of CO, which is presented in Figure 5b for all applied potentials and temperatures. Importantly, it shows that the TOF of CO formation increases from 20 to 50 °C at −0.6 *V*_RHE_ and −0.8 *V*_RHE_. However, at −1.0 *V*_RHE_, it reaches the maximum at 40 °C and then drops at 50 °C. Thus, increasing TOF with increasing temperature indicates that the rate of the CO_2_-to-CO conversion is additionally influenced by the temperature. Also, the TOF of H_2_ (see Figure S7) increases with temperature for all applied potentials, suggesting that the composite is more active for H_2_ formation at elevated temperatures.

The production rates of CO and H_2_ are presented in Figure 5c,d. It shows that with increasing temperature, production rates of both gases increase at −0.6 *V*_RHE_ and −0.8 *V*_RHE_. At –1.0 *V*_RHE_, the rate of CO formation increases from 20 to 40 °C, while that of H_2_ increases from the lowest to the highest temperature. This similar trend is observed also for the TOF, FE, and *j*_p_ of CO and H_2_ formation. The production rates of CO at −1.0 *V*_RHE_ at 20 and 40 °C are comparable with the most active nanoporous Au catalyst reported (see Table S1).

**Figure 6:**
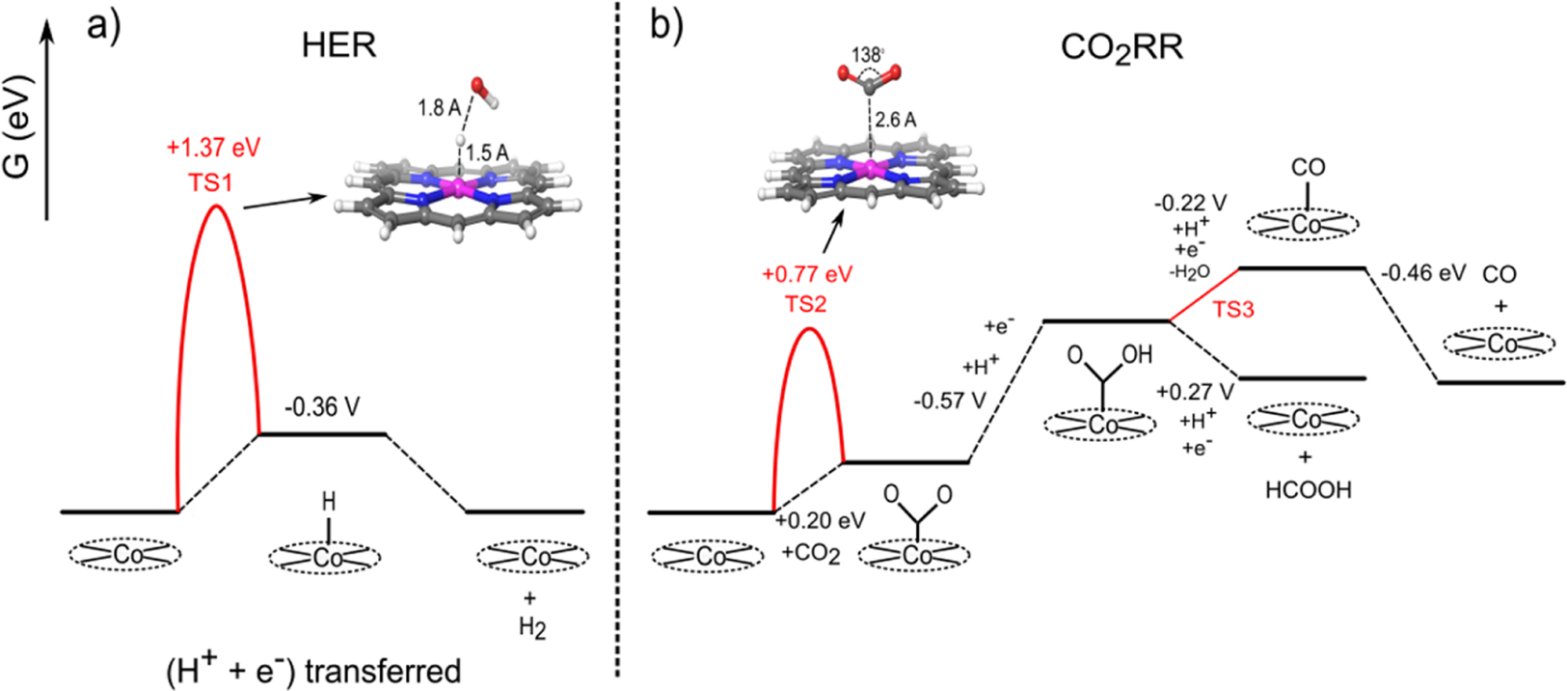
Gibbs free-energy profiles of (a) HER and (b) eCO_2_R on Co(0)TPP (starting point) at pH = 7; potentials are versus RHE.

The influence of temperature on the electrochemical reaction rates of CO and H_2_ formation is evaluated by Arrhenius activation energy (*E*_a_) calculation. In relation to this, potential vs apparent *E*_a_ (Figure S9) was calculated to understand the influence of potential on the energy barrier for the rate-determining step of CO and H_2_. When comparing the CO (Figure S8a) and H_2_ formation (Figure S8b) rates, temperature appears to influence less in the former. The *E*_a_ for H_2_ formation is 70.2 kJ/mol at −0.6 *V*_RHE_, 84.2 kJ/mol at −0.8 *V*_RHE_, and 58.0 kJ/mol at −1.0 *V*_RHE_. A high *E*_a_ for H_2_ formation at all studied potentials (see Figure S9) is expected in a CO_2_ environment as it depends on the magnitude of the energy barrier for the rate-limiting step and H_2_O molecule reorganization.^62,63^ For CO formation, *E*_a_ is 14.2 kJ/mol at −0.6 *V*_RHE_, 43.0 kJ/mol at −0.8 *V*_RHE_, and 10.8 kJ/mol at −1.0 *V*_RHE_*.* The highest *E*_*a*_ of CO ca 43 kJ mol^–1^ is observed for −0.8 *V*_RHE_, and considering this, the rate-limiting step is postulated to involve a chemical step.^64^ The lowest *E*_a_ at −1.0 *V*_RHE_ is understandable from the DFT results (see Figure 6b), indicating that the reaction path is barrierless at this potential. However, the explanation for *E*_a_ at −0.6 *V*_RHE_ remains currently unclear.

### 3.4 Understanding CO Formation Selectivity Trends by DFT

The experimental results show that the CoTPP/MWCNT composite is highly selective for CO production at more negative potentials. Furthermore, the H_2_/CO ratio of syngas is increased when the temperature increases. To understand the origin of these trends, DFT modeling is employed. In general, the eCO_2_R starts with the direct adsorption of CO_2_ at the catalyst surface from where it is transformed to either CO or more reduced products through stepwise protonation.^65^ An alternative to this direct reduction path is the reaction of CO_2_ with a cobalt hydride (Co–H) intermediate to form formic acid.^66^

The cobalt hydride is typically formed through the discharge of a proton or H_2_O to form a Co–H hydride (Volmer reaction). Naturally, this intermediate can also react further to produce hydrogen through the reaction with a second proton or water molecule (Heyrovsky reaction). Due to the very low concentration of H_3_O^+^ of only 10^–7^ mol/L under the reaction conditions (pH = 7), H_2_O is the most likely proton source under experimental conditions.^67^

Depending on the potential, the reactions will proceed either over Co(II), Co(I), or Co(0). Co(I) is only relevant at the potential of −0.6 *V*_RHE_. With decreasing the potential to −0.8 *V*_RHE_ and −1.0 *V*_RHE_, Co(I) can be further reduced to Co(0). Interestingly, Co(II)–H is not stable under the reaction conditions. This can be realized by the comparison of the HER and the CO_2_RR on Co(II). The Volmer reaction to form a Co(II)–H species through a PCET requires a redox potential of −0.84 *V*_RHE_. Thus, it cannot occur in the potential range where Co(II) would be stable. Furthermore, the formed hydride is a very strong acid with a *p*K_a_ of −14 and will therefore readily dissociate into the bare CoTPP complex and a proton. The presence of a strongly acidic hydride might appear surprising at first glance. However, it is well established in organometallic chemistry that pKa of a hydride can be comparable to that of strong acids in the presence of strongly electron-withdrawing ligands or metal centers in higher oxidation states.^68^ Accordingly, any follow up reactions to form H_2_ or formic acid are blocked. Similarly, CO_2_ is also unstable on Co(II)TPP and readily desorbs since it cannot provide the necessary electrons to convert linear CO_2_ to a bent Co–COO carboxylate intermediate. Hence, both the eCO_2_R and the HER are blocked on Co(II)TPP.

On Co(I)TPP, the redox potential for forming the Co(I)–H hydride is −0.61 *V*_RHE_ and thus, in principle, feasible at all considered potentials. However, this reaction is, identical to Co(II), blocked by the rather high acidity of the “hydride” proton, which with a *p*K_a_ of 4 will readily dissociate under reaction conditions, for example, a pH of approximately 7. Note that proton-transfer reactions are subject to only a very minor activation barrier of the order of 0.1 eV^69^ and thus can be expected to outcompete any follow-up reaction. Furthermore, the reaction is subject to a very high activation barrier of 1.7 eV for forming Co(I)–H from water (Figure S1), thus effectively blocking the HER and the eCO_2_R to formic acid through the hydride route.

Co(0) finally forms with a *p*K_a_ of 10, a stable hydride under the reaction conditions. Forming this hydride requires a potential of −0.36 *V*_RHE_ (Figure 6a) and can therefore be easily facilitated at the potentials of −0.8 *V*_RHE_ and −1.0 *V*_RHE_, where at higher temperatures significant amounts of H_2_ are observed. However, this reaction is again blocked by a very high activation barrier of 1.4 eV for water as a proton source (Figure 6a). It must be cautioned that this activation can be subject to significant errors of ca. ±300 meV, owing to neglecting of explicit water molecules in the model. Furthermore, H_3_O^+^ is a more reactive proton source,^67^ and it is available in very small concentrations. Thus, the formation of Co(0)-H may become feasible at rather low reaction rates. This in turn would, in line with experimental evidence (see Figure 4b,c), enable the formation of minor amounts of hydrogen. Naturally, also increasing the temperature will increase the rate of hydrogen formation further. Additionally, the Co(0)-H path for the eCO_2_R to form formic acid is, owing to the consistently low activation barrier,^70^ in principle, also possible but is unlikely, owing to the very low concentrations of CO_2_ in the electrolyte.

The unfavorable formation of hydrides leaves the direct reaction of CO_2_ with cobalt as the only viable reaction route. This reaction is initialized by the direct adsorption of CO_2_ at the active site to form a Co–COO carboxylate (Figure 6b**)**. As a result of the significant loss of entropy when binding a gas phase CO_2_ to the catalysts, the direct CO_2_ adsorption on Co(0)TPP and Co(I)TPP are found to form Co–COO bonds which are slightly endergonic by +0.20 and +0.45 eV, respectively (Figure 6b for Co(0) and Figure S1 for Co(I)). Hence, increasing the temperature will increase the entropy costs for this reaction even further and thus render this reaction step less likely. Thus, the selectivity will, in line with experimental evidence, shift toward increased hydrogen in the gas mixture (see Figure 4a,b,c). The reactions are subject to rather high activation barriers of +0.77 eV for Co(0)TPP and +0.67 eV for Co(I)TPP (TS2 in Figures 6b and TS S1). This high barrier is most likely due to the energy cost associated with bending of the linear CO_2_ molecule in a carboxylate-like geometry with an O–C–O bond angle of roughly 130°. Carboxylate formation is followed by the protonation to Co–COOH. Again, water serves as a proton source for this reaction. The redox potential of the reaction is −0.60 V_RHE_ on Co(I)TPP. However, the obtained acid has a *p*K_a_ of only 4 and thus readily dissociates in the pH-neutral electrolyte. Moreover, the PCET reduction of Co(I)TPP to form Co(0)–COOH requires a comparable potential of −0.57 *V*_RHE_. This Co(0)-COOH species has a high *p*K_a_ value of 10 which renders it stable toward the deprotonation under reaction conditions. Thus, the only route forward for Co(I)–COO is the reduction through the addition of an electron, leading to the Co(0)–COO route which requires a potential of −0.71 *V*_RHE_. It is important to note that Co–COOH is a crucial intermediate in the pathway since two different products can be obtained from it. A second proton can electrochemically attack the OH group in Co(0)-CCOH and eliminate the water molecule, leading to the formation of Co(0)–CO. The reaction requires a potential of only −0.22 V and is surprisingly also barrierless after activation through protonation as evident from the PES scan (Figure 6b). Considering a moderately strong binding energy, CO can be released as the final product, for example, the final release is endergonic by 0.46 eV. Again, the second possible pathway is the attack of the proton on the carbon atom of Co–COOH in an electrochemical reaction on Co(0)TPP. This would result in the breaking of the Co(0)-C bond and the release of HCOOH. Energetically, this reaction is highly favorable and requires a redox potential of +0.27 eV. However, since the carbon atom in the Co(0)–COOH intermediate is electron poor and additionally a Co(0)–C bond also needs to be broken, the proton attack will result in a significant energy barrier. This in turn renders the formation of formic acid, in line with the experimental evidence, unfavorable.

Overall, the high selectivity toward CO_2_ reduction to form CO is the result of the very high activation barriers associated with the formation of the Co(0)–H hydride intermediates which could facilitate hydrogen evolution, while CO_2_ adsorption, which is the rate-determining step toward CO formation, requires a significantly lower barrier of the order of 0.7 eV. In line with experimental evidence, our computations suggest an onset potential of −0.6 *V*_RHE_. Thus, CO_2_ reduction would in principle be possible at all experimentally considered potentials. However, in reality, it is blocked by the almost complete absence of Co(I) and Co(0) at the lowest overpotential of −0.6 *V*_RHE_, where the experiment also shows negligible partial current densities (Figure 4a). For −0.8 *V*_RHE_ and −1.0 *V*_RHE_, the active Co(0)TPP complex is finally available, resulting in the experimentally observed formation of significant amounts of CO (Figure 4b,c).

## 4 Conclusions

In this work, we have investigated the temperature influence on eCO_2_R on a CoTPP/MWCNT composite in a flow cell and discovered that temperature can be a parameter which controls the ratio of H_2_ and CO in the formed syngas. This finding is beneficial for industrial applications where different H_2_/CO ratios are required to produce various important chemicals and fuels. Importantly, when the temperature is increased, the selectivity for CO formation decreased accompanied with increased selectivity for H_2_ at all studied potentials. Although the selectivity for CO production decreases with increasing temperature, the partial current density increases, suggesting that higher temperatures accelerate the CO production by increasing the reaction rate. In addition to the experimental work, DFT modeling has been used to find the origin of high selectivity of the composite for CO formation in these experimental conditions and identifying the key oxidation states of the active site, for example, at −0.6 *V*_RHE_, the mostly dominating active site is Co^2+^ but at −0.8 and −1.0 *V*_RHE_, it is Co^°^. We believe that this investigation of the temperature-controlled syngas production would contribute an alternate way for the production of syngas.

## Supporting Information Available

Computation details, electrode preparation, SEM analysis, XRD analysis, CV analysis, product distribution with time, turnover frequency for H_2_, CO production-rate comparison data, ln*j*_p_ versus time graph, and potential versus activation energy graph (PDF)
