# Supplementary material for: Temperature-Controlled Syngas Production via Electrochemical CO2 Reduction on a CoTPP/MWCNT Composite in a Flow Cell
Source: ACS Appl Energy Mater. 2022 Dec 22;6(1):267–77. doi: 10.1021/acsaem.2c02873 (PMC9832436; doi:10.1021/acsaem.2c02873)
Supplement: Supplementary file 1 — ae2c02873_si_001.pdf [file ae2c02873_si_001.pdf]

Supporting Information

Temperature Controlled Syngas Production via Electrochemical CO_2 Reduction on a CoTPP/MWCNT composite in a Flow Cell

M. Noor Hossain[a], Reza Khakpour[a], Michael Busch[a], Milla Suominen[a], Kari Laasonen[a], Tanja Kallio[a,*]

[a] Department of Chemistry and Materials Science, Aalto University School of Chemical Engineering, P.O. Box 16100, FI-00076 AALTO, Finland

*corresponding author: tanja.kallio@aalto.fi

Computational details

The Gibbs free energies were calculated by standard method in 16 Rev C.01 package using frequency calculation:

$$G = E_{tot} + E_{ZPE} - TS$$

Here, E_{tot} represents the total energy of the system, E_{ZPE} shows zero-point energy, T corresponds to constant temperature of the system (298.15 K), and S accounts for Entropy of the system. Consequently, Gibbs free energy difference of a reaction was computed by:

$$\Delta G_{(reaction)} = \Delta G_{(product)} - \Delta G_{(reactant)}$$

E.g.

$$CoTPP + CO_2 \longrightarrow CO_2\text{-}CoTPP$$

$$\Delta G_{(reaction)} = \Delta G_{(CO2\text{-}CoTPP)} - \Delta G_{(CoTPP)} - \Delta G_{(CO2)}$$

$$CO_2\text{-}CoTPP + H^+ + e^- \longrightarrow COOH\text{-}CoTPP$$

$$\Delta G_{(reaction)} = \Delta G_{(COOH\text{-}CoTPP)} - \Delta G_{(H^+ + e^-)} - \Delta G_{(CO2\text{-}CoTPP)}$$

Note: Bare CO_2, H_2, and CO molecules were optimized in gas phase while for dissolved reactants and intermediates, the structures were optimized using SMD solvation model.

Standard hydrogen electrodes (SHE)[1] were utilized as reference electrodes for proton coupled electron transfer (PCET) steps.

$$H^+ + e^- \longrightarrow 0.5{*}H_2\,(g) \qquad E^0 = 0\ V$$

$$G\,(H^+ + e^-) = 0.5{*}G\,(H_2,\ gas)$$

Where, G (H_2, gas) represents the Gibbs free energy of H_2 in gas which was calculated by DFT. To consider the pH dependence, PCET steps were divided into electron transfer (ET) and proton transfer (PT). Using the ‘effective absolute potential method, Gibbs free energy of ET steps were calculated.[2] Considering Gibbs free energy of their reduction reactions, the standard redox potentials were calculated based on the equation below.[3]

$$E^0 = -\Delta G_{(reaction)}/nF$$

Where n is number of transferred electrons, F is Faraday constant, and $\Delta G_{(reaction)}$ is the Gibbs free energy difference of the reduction reaction. At pH=0, E^0_{SHE} is equivalent to E^0_{RHE}. All

redox potentials were converted into reversible hydrogen electrodes (RHE) reference.[4] Regarding, the pH rise was also considered:

$$E^0_{RHE} = E^0_{RHE\ (PH=0)} + 0.059*pH$$

Owing to the SMD solvation model error for different charge states of reactant and product, the pK_a of PT steps were computed based on the experimental value of HCOOH dissociation as a reference (instead of the SHE model):

$$pK_a = \Delta G_{(isodesmic)} / RTln(10) + pK_{a\ (ref)}$$

where $\Delta G_{(isodesmic)}$ is the reaction energy of the proton transfer from the compound of interest (AH) to the corresponding base of the reference acid:

$$AH + B \longrightarrow A^- + BH$$

Also, $pK_{a(ref)}$ is the pK_a of our reference (formic acid).[5–8] A linear scaling approach was used to correct the shortcomings of the SMD approach for predicting accurate pK_a values based on earlier work [Busch, Ahlberg Laasonen, submitted]. In accordance with this work, formic acid was used as the reference compound.

$$pK_a(scaled) = (0.49*pK_a(DFT)) + 3.2$$

In this regard, it is also necessary to correct the ΔG of pure ET steps. Due the fact that $\Delta G_{(PT)\ (scaled)} + \Delta G_{(ET)}$ must be equal with $\Delta G_{(PCET)}$ and $\Delta G_{(PCET)}$ is accurate enough in SMD solvation model calculation, it is possible to indirectly scale $\Delta G_{(ET)}$ value:

$$\Delta G_{(ET)} = \Delta G_{(PCET)} - \Delta G_{(PT)\ (scaled)}$$

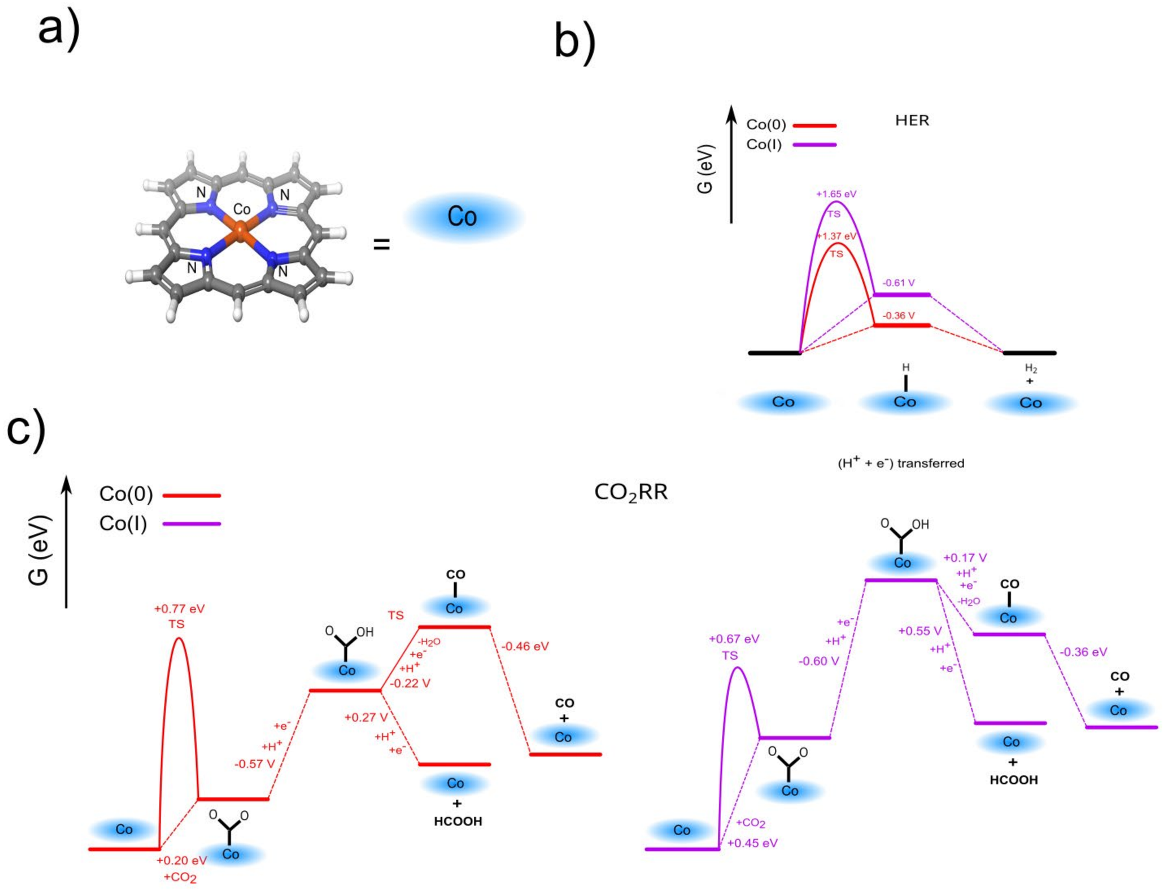

Figure S1: (a) the CoTPP model system for CoTPP/MWCNT catalyst, (b) HER and (c) CO2RR energy profiles on Co(0)TPP and Co(I)TPP at pH=7.

Experimental Details

Scanning Electron microscopy

The SEM images of fresh electrode was taken by using Zeiss Sigma VP equipped with a Schottky field emission gun (FEG). The used electrode was investigated by Tescan Mira3.

X-ray diffraction

The XRD profile of fresh, used composite electrodes, pristine CoTPP and MWCNT was evaluated by using X-ray diffraction (Pananalytical X´pert pro MPD alpha 1) with Cu Kα x-ray source at 45 KV and 40 mA. The scanning angle was 10º to 80º with scan rate of 1º / min.

MWCNT Electrode Preparation

15.5 mg of MWCNT was weighed and dispersed in a 4 mL of pure ethanol. Then, 5 wt-% Nafion (Sigma-Aldrich) solution was added in the ink to obtain 30 wt-% Nafion ionomer content. The ink was sonicated for ca. 30 minutes and then stirred for 24 h and 1 mL of the ink was sprayed on 4 cm^2 microporous carbon paper (Sigracet 25 BC) gas diffusion electrode (GDE) by employing an air brush. Before deposition of the composite, the GDE was cleaned with acetone and dried in a vacuum oven for 2 h at 60-70°C and weighed. After spraying the ink, the electrode was dried again in a vacuum oven at 60-70°C for 1.5 h.

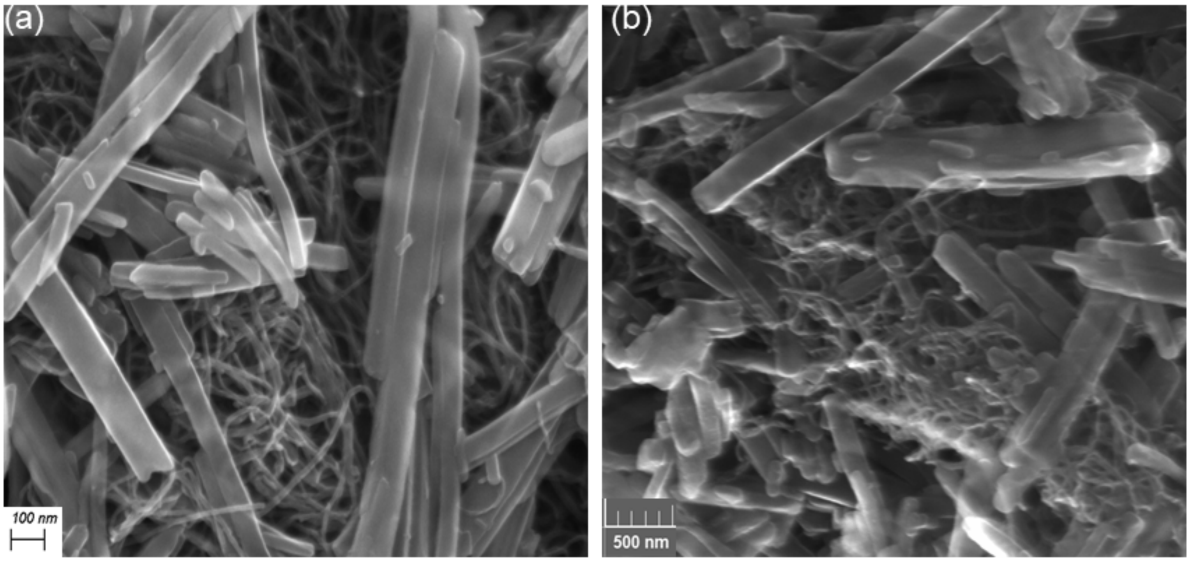

Figure S2: Scanning electron microscopy (SEM) images of CoTPP/MWCNT composite electrodes a) Fresh and b) Used (at -1.0 V_{RHE}).

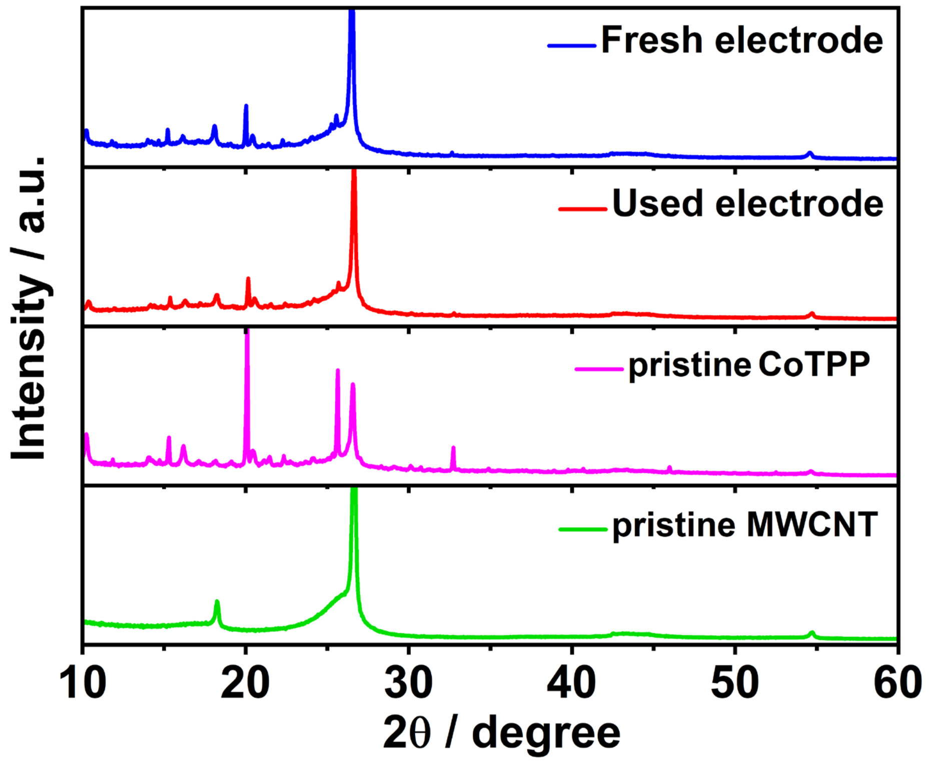

Figure S3: XRD patterns of a fresh, used (-1.0 V_{RHE}) CoTPP/MWCNT electrodes, pristine CoTPP and MWCNT.

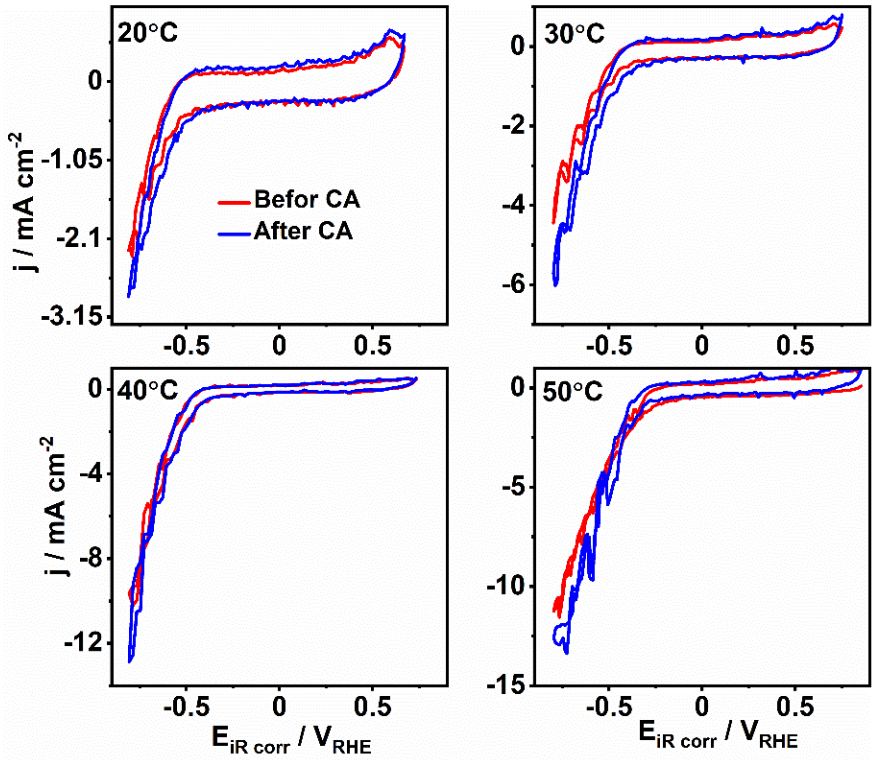

Figure S4: CVs before and after the CA measurement at -0.8 V_{RHE}.

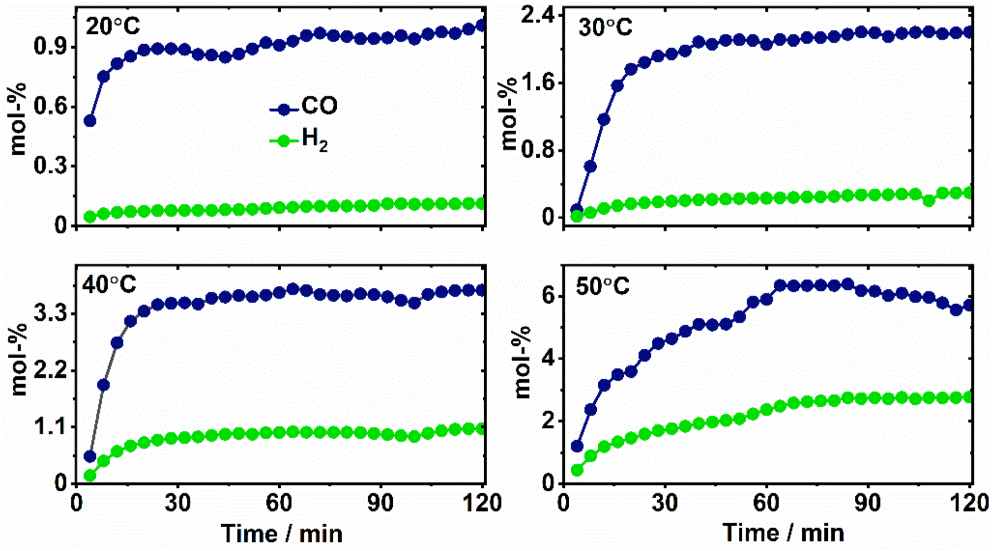

Figure S5: Major product distribution curves as a function of time at -0.8 V_{RHE} suggest that the mol-% of the products change with time due to increased wettability of the electrode during the measurements.

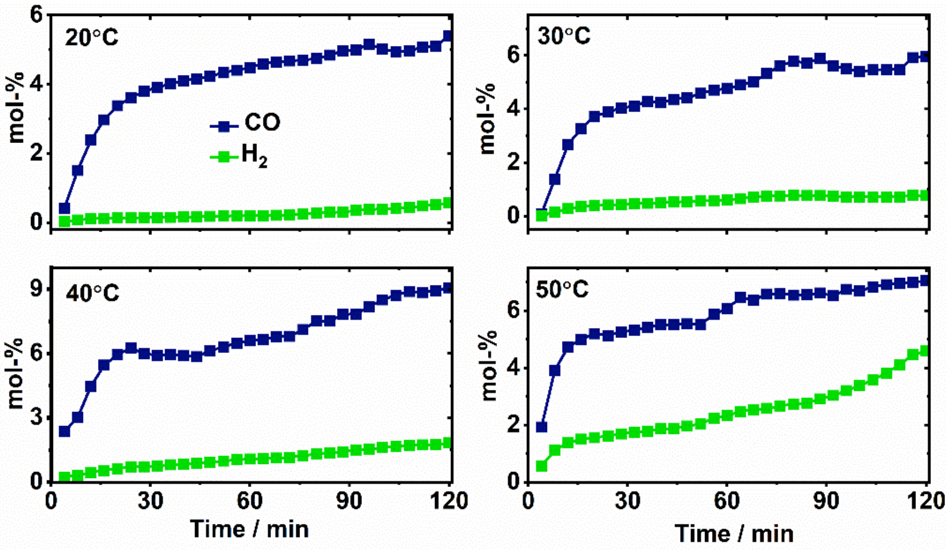

Figure S6: Major product distribution curves as a function of time at -1.0 V_{RHE} suggest that the mol-% of the products change with time due to increased wettability of the electrode during the measurements.

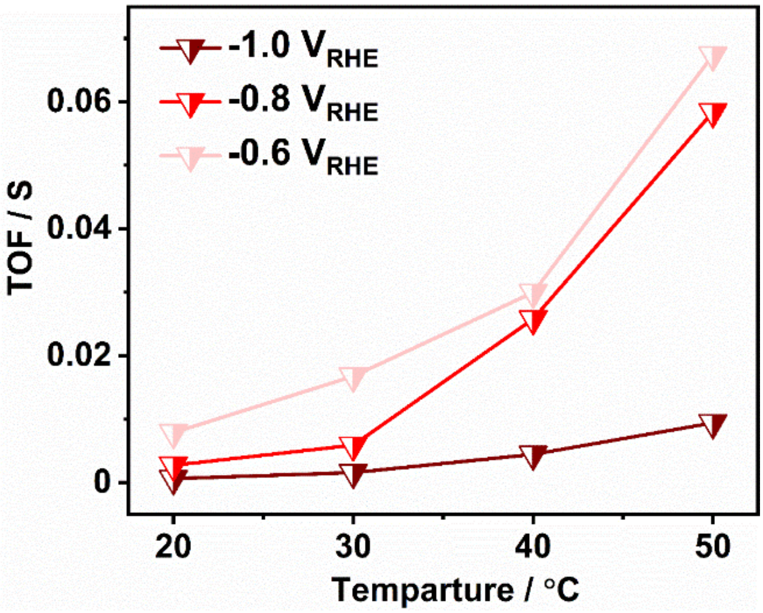

Figure S7: Turn of over frequency of H_2 production.

Table S1: Comparison of the CO production rates with various earlier reported CO selective electrocatalyst materials.

Catalysts	Temperature (°C)	Applied potential	FE (%)	Production rate	Ref:
CoTPP/MWCNT	20	-1.0 V_{RHE}	98	186 µmol cm^{-2} h^{-1}	This work
CoTPP/MWCNT	40	-1.0 V_{RHE}	91	280 µmol cm^{-2} h^{-1}	This work
CoTPP/MWCNT	25	-1.35 V_{SCE}	91	416 µmol	9
Fe-Porphyrin	25	-1.3 V_{NHE}	54	15.3 µmol cm^{-2} h^{-1}	10
Co^{2+}(Cl)/MWCNT	25	-1.1 V_{NHE}	89	15 µmol h^{-1}	11
NP Au	25	-0.6 V_{RHE}	95.8	300 µmol cm^{-2} h^{-1}	12
Cu-rGO	25	-0.6 V_{RHE}	40	20 µmol cm^{-2} h^{-1}	13
Cu 60-80 nm	25	2.5 $V_{Ag/AgCl}$	5	17 µmol m^{-2} s^{-1}	14
Cu NPs/CP	25	1.8 $V_{Ag/AgCl}$	21	12 µmol m^{-2} s^{-1}	15

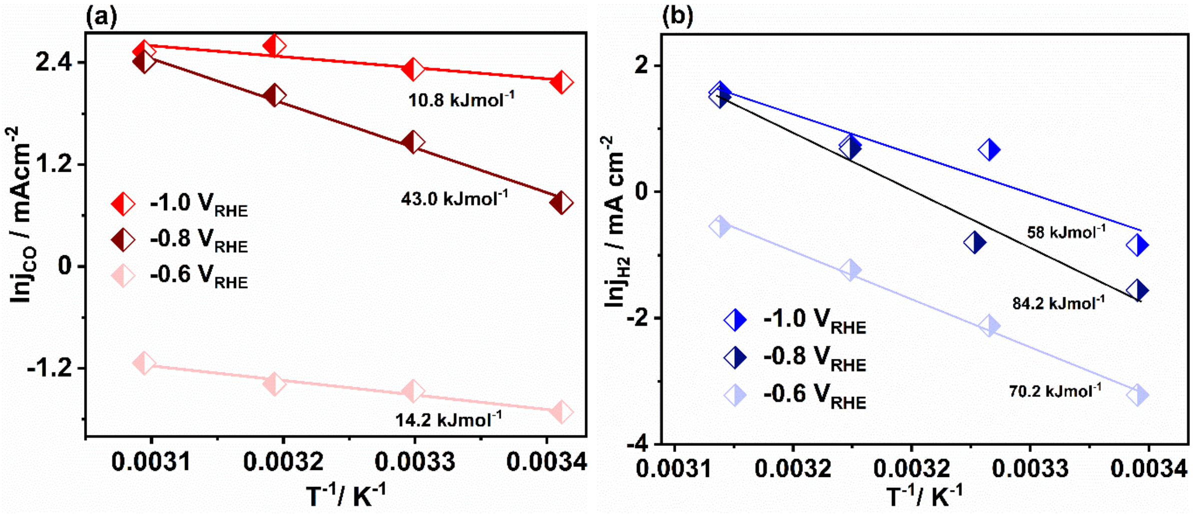

Figure S8: $\ln j_p$ vs 1/T curve for a) CO and b) H_2.

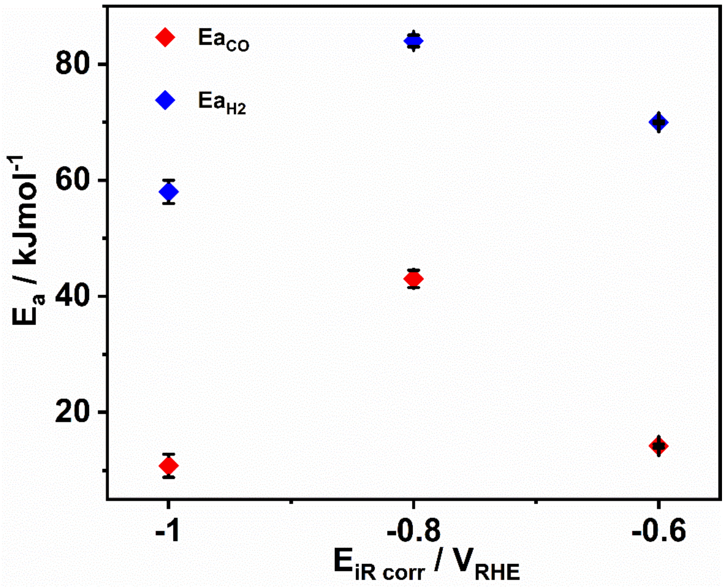

Figure S9: Potential vs. activation energy curve for CO and H_2.

References

(1) The Absolute Electrode Potential: An Explanatory Note (Recommendations 1986). *J. Electroanal. Chem. Interfacial Electrochem.* **1986**, *209* (2), 417–428. https://doi.org/10.1016/0022-0728(86)80570-8.
(2) Busch, M.; Laasonen, K.; Ahlberg, E. Method for the Accurate Prediction of Electron Transfer Potentials Using an Effective Absolute Potential †. *Phys. Chem. Chem. Phys* **2020**, *22*, 25833. https://doi.org/10.1039/d0cp04508j.
(3) Bard, A. J., Faulkner, L. R. *Electrochemical Methods: Fundamentals and Applications, 2nd Edition | Wiley*, 2nd ed.; Wily , 2001.
(4) Staehler, M.; Wipperman, K.; Stolten, D. Instabilities of the Reversible Hydrogen Reference Electrode in Direct Methanol Fuel Cells.
(5) Ho, J.; Coote, M. L. PK a Calculation of Some Biologically Important Carbon Acids-An Assessment of Contemporary Theoretical Procedures. https://doi.org/10.1021/ct800335v.
(6) Ho, J.; Coote, M. L. A Universal Approach for Continuum Solvent PK a Calculations: Are We There Yet? https://doi.org/10.1007/s00214-009-0667-0.
(7) Casasnovas, R.; Ortega-Castro, J.; Frau, J.; Donoso, J.; Mu~, F. Theoretical PK a Calculations With Continuum Model Solvents, Alternative Protocols to Thermodynamic Cycles. https://doi.org/10.1002/qua.24699.
(8) Ho, J. Predicting PKa in Implicit Solvents: Current Status and Future Directions*. *Aust. J. Chem.* **2014**, *67* (10), 1441–1460. https://doi.org/10.1071/CH14040.
(9) Hu, X.-M.; Rønne, M. H.; Pedersen, S. U.; Skrydstrup, T.; Daasbjerg, K. Enhanced Catalytic Activity of Cobalt Porphyrin in CO $_2$ Electroreduction upon Immobilization on Carbon Materials. *Angew. Chemie Int. Ed.* **2017**, *56* (23), 6468–6472. https://doi.org/10.1002/anie.201701104.
(10) Hod, I.; Sampson, M. D.; Deria, P.; Kubiak, C. P.; Farha, O. K.; Hupp, J. T. Fe-Porphyrin-Based Metal-Organic Framework Films as High-Surface Concentration, Heterogeneous Catalysts for Electrochemical Reduction of CO2. *ACS Catal.* **2015**, *5* (11), 6302–6309. https://doi.org/10.1021/acscatal.5b01767.
(11) Aoi, S.; Mase, K.; Ohkubo ab, K.; Fukuzumi, S. Open Access Article. *Chem. Commun* **2015**, *51*, 10226. https://doi.org/10.1039/c5cc03340c.
(12) Hossain, M. N.; Liu, Z.; Wen, J.; Chen, A. Enhanced Catalytic Activity of Nanoporous Au for the Efficient Electrochemical Reduction of Carbon Dioxide. *Appl. Catal. B Environ.* **2018**, *236*, 483–489. https://doi.org/10.1016/J.APCATB.2018.05.053.
(13) Hossain, M. N.; Wen, J.; Chen, A. Unique Copper and Reduced Graphene Oxide Nanocomposite toward the Efficient Electrochemical Reduction of Carbon Dioxide. https://doi.org/10.1038/s41598-017-03601-3.
(14) Merino-Garcia, I.; Albo, J.; Solla-Gullón, J.; Montiel, V.; Irabien, A. Cu Oxide/ZnO-Based Surfaces for a Selective Ethylene Production from Gas-Phase CO2 Electroconversion. *J. CO2 Util.* **2019**, *31* (March), 135–142. https://doi.org/10.1016/j.jcou.2019.03.002.
(15) Merino-Garcia, I.; Albo, J.; Irabien, A. Productivity and Selectivity of Gas-Phase CO2 Electroreduction to Methane at Copper Nanoparticle-Based Electrodes. *Energy Technol.* **2017**, *5* (6), 922–928. https://doi.org/10.1002/ente.201600616.
